# An ecological model of adaptation to displacement: individual, cultural and community factors affecting psychosocial adjustment among Syrian refugees in Jordan

**DOI:** 10.1017/gmh.2018.30

**Published:** 2018-12-20

**Authors:** Ruth Wells, Catalina Lawsin, Caroline Hunt, Omar Said Youssef, Fayzeh Abujado, Zachary Steel

**Affiliations:** 1 Clinical Psychology Unit, School of Psychology, University of Sydney, Sydney, Australia; 2 Trauma and Mental Health Unity, School of Psychiatry, University of New South Wales, Sydney, Australia; 3 St John of God Health Care, Richmond Hospital, Sydney, Australia; 4 Rush University Medical Centre, Chicago, USA; 5 Syria Bright Future, Amman, Jordan

**Keywords:** Ecological, psychosocial, Syria, refugee mental health, qualitative, other

## Abstract

**Background.:**

There is a need for ecological approaches to guide global mental health programmes that can appropriately address the personal, family, social and cultural needs of displaced populations. A transactional ecological model of adaptation to displacement was developed and applied to the case of Syrian refugees living in Jordan.

**Methods.:**

Syrian and Jordanian psychosocial workers (*n* = 29) supporting the Syrian refugee community in Jordan were interviewed in three waves (2013–2016). A grounded-theory approach was used to develop a model of key local concepts of distress. Emergent themes were compared with the ecological model, including the five ADAPT pillars identified by Silove (2013).

**Results.:**

The application of the ecological concept of niche construction demonstrated how the adaptive functions of a culturally significant concept of dignity (*karama*) are moderated by gender and displacement. This transactional concept brought to light the adaptive capacities of many Syrian women while highlighting the ways that stigma may restrict culturally sanctioned opportunities for others, in particular men. By examining responses to potentially traumatic events at the levels of individual, family/peers, society and culture, adaptive responses to environmental change can be included in the formulation of distress. The five ADAPT pillars showed congruence with the psychosocial needs reported in the community.

**Conclusions.:**

The transactional concepts in this model can help clinicians working with displaced people to consider and formulate a broader range of causal factors than is commonly included in individualistic therapy approaches. Researchers may use this model to develop testable hypotheses.

More than half a million people have lost their lives in the current Syrian conflict (SOHR, 2018) and more than 11 million have been displaced (UNHCR, 2018). There are over 600 000 Syrian refugees living in Jordan (UNHCR, 2018) mostly in the host community, where services to address their needs may be limited (Murshidi *et al*. 2013). Many have been subjected to an array of potentially traumatic events (PTEs) (Hassan *et al*. 2016) known to elevate the risk of mental health problems (Steel *et al*. 2009), while the stresses of living in displacement have been demonstrated to intensify these issues (Wells *et al*. 2016*a*). We hope that an ecological approach to understanding distress within local meaning systems, and in response to local challenges, may help mental health practitioners respond to this crisis in ways that can support existing coping mechanisms.

Our recent systematic review of psychosocial concerns reported by Syrian refugees living in Jordan (Wells *et al*. 2016*a*) highlighted how threats to safety (such as physical or sexual abuse, inadequate housing, exploitation and financial strain) were commonly reported, along with discrimination and perceived injustice. These environmental stressors lead to psychosocial stressors, such as loss of role, forced changes to identity and loss of social support. These combined with the impact of PTEs and distress symptoms to increase family violence, further disrupting interpersonal functioning. In the model derived from this systematic review, we describe the impact of displacement stressors as cumulative and interactive and recommend that psychosocial interventions focus on addressing the psychosocial impacts of environmental stressors by promoting access to resources, such as volunteering, employment, social support or skills acquisition, which is in line with international norms for psychosocial programming in crisis settings (IASC, 2007). That is, as outlined by Silove in the ADAPT model, providing opportunities to develop new roles, identities, existential meaning, bonds or justice (Silove, 2013) can support individual and community resilience.

Theoretical models sensitise researchers and clinicians to focus on specific issues. In this paper, we highlight the contribution of political, social and ecological forces which shape adaptation and wellbeing in displacement (Hess, 2005) and argue for a theoretical perspective which analyses how individuals and communities adapt to new ecological contexts (Miller & Rasco, 2004). Our aim is to advance an ecological model which examines the reciprocal relationships between individual psychological functioning and environmental factors pre-, during and post-displacement. In the last two decades, a greater emphasis on the social and cultural causes of distress (Summerfield, 1999; Hobfoll, 2001) and cultural adaptation (Bolton, 2001; Bolton & Tang, 2004) have contributed to international practice guidelines which are more attentive to the perceived needs and social and economic circumstances of communities affected by crisis (IASC, 2007; Lund *et al*. 2012). Often it is the daily stressors experienced in displacement that are the most salient concerns for affected communities (Wells *et al*. 2016*a*, *b*) and mediate distress caused by PTEs (Miller & Rasmussen, 2010). We require theoretical and methodological tools to explore how intersecting environmental factors impact on adaptive functioning (Miller & Rasmussen, 2014).

## Ecological understandings of distress: social resources for adaptation

An ecological/transactional model can highlight an individual's role in responding to environmental challenges commonly described as risk factors (e.g. human rights violations). Such models can explore how the structural properties of the displacement environment produce different adaptive role functions (Kelly, 1966). Bronfenbrenner (1979) proposed an ecological theory of human development which conceptualised individuals as nested within multiple interacting systems. This includes intra-individual, interpersonal and larger social systems, with subsequent adaptations (Earls & Carlson, 2001; Tol *et al*. 2008). Drozdek, in applying these systems to refugee mental health, described the nested layers as intra-individual, family/peers, society and culture (Drozdek, 2015).

Ecological systems are transactional in the sense that individuals can influence the nature of their social contexts, while also being subject to social constraints (Joyce, 2002). By describing individual human agency in response to social systems (Altorki, 2015), transactional models are useful for describing how people may use resources for resilience in the face of adversity (Cicchetti, 2010; Kohrt *et al*. 2015). Niche construction (Kendal *et al*. 2011) is a transactional concept which describes how changes we make to our environment can change how the environment influences us. We develop a niche (i.e. access to resources), promoting adaptation and providing ecological inheritance, often in the form of culture. Kelly (1966) argued that more random environments, where resource availability is unpredictable, will lead to a broader niche (i.e. flexibility to adapt to diverse environments). Ethnographic research in Syria in the 1990s demonstrated how Syrians developed a set of conversational norms that allowed them to speak relatively freely within everyday discourse without putting themselves in danger of arrest in an autocratic system (Wedeen, 2015). These conversational norms may be seen as resources employed for adaptation.

The way we interact with resources is a key component of adaptation and resilience. Resources relevant to displaced people may include financial savings, personal resilience, family cohesion, qualifications, language skills and an ability to act within the cultural norms of the host society. Ryan *et al*. (2008) argue that resources should be considered at pre-, during and post-migration phases. Resources may be coerced (e.g. paying money to flee conflict) or underutilised (e.g. unable to work in the host country). Some resources may not be lost, rather their value changes with a change in environment (e.g. qualifications not recognised in new countries). In addition, people's ability to take advantage of resources may be undermined by the nature of traumatic experience. Human rights violations and PTEs are commonly associated with depression (Mollica *et al*. 1993); anger (Brooks *et al*. 2011); and interpersonal and emotion regulation difficulties (Nickerson *et al*. 2014) as well as post-traumatic stress disorder (Steel *et al*. 2009) and resilience (Hijazi *et al*. 2014). The ADAPT model (Silove, 2013) takes this into account, along with the breakdown in social systems, to consider adaptive functioning across five adaptive systems. These include safety and security; bonds and networks, justice; roles and identities; and existential meaning. The model provides a framework to examine how the interactions between these systems may lead to a wide range of presentations through the meaning we ascribe to salient aspects of our lives (Tay & Silove, 2017).

## A model of adaptive processes in the context of displacement

**Fig. 1.**
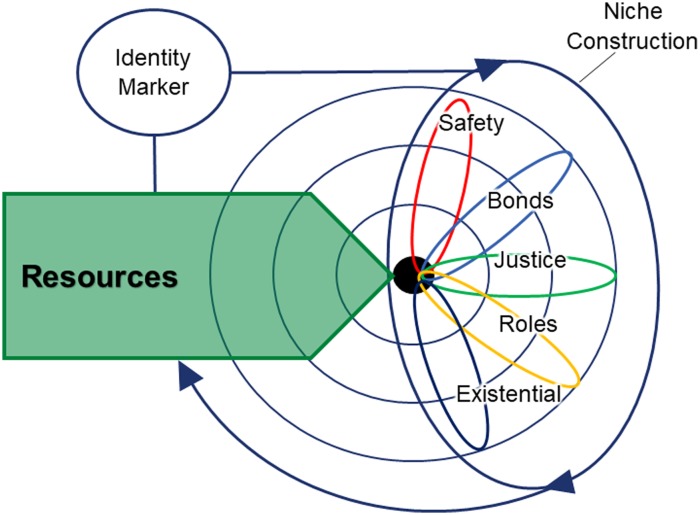
The process of adaptation to the environment through niche construction: the individual nested in a social world. Note: The model depicts the process whereby resources are invested in modifying the environment, via the five adaptive systems, which in turn affect available resources. The concentric circles represent the individual nested in the layers of family/peers, society and culture (Bronfenbrenner, 1979; Drozdek, 2015). The five adaptive systems (Silove, 2013) are engaged by the individual in relation to the layers of the environment to promote wellbeing. Overarching these systems is the process of niche construction, whereby resources are invested in modifying the environment in order to promote wellbeing. This reciprocal process effects access to future resources. In addition, the process by which resources are invested is moderated to identity markers including gender, age, social status, class, ethnicity, disability and previous history of PTEs, affecting the kinds of behaviours engaged for niche construction.

We now describe an ecological model to explore how adaptation is impacted by displacement. Figure 1 depicts the individual nested in the layers of family and peers, society, and culture (Bronfenbrenner, 1979; Drozdek, 2015). The overarching process of niche construction operates to promote adaptation across the layers of the environment. This is further delineated into the five adaptive systems identified by Silove (2013) above. The individual uses these adaptive systems to operate within the nested layers of the environment. Resources [personal, material, social and cultural (Hobfoll, 2001)] are invested in order to promote adaptation. Through the process of niche construction, invested resources modify the environment, producing future resources. This process is moderated by the impact of identity markers, such as gender, history of PTEs, age, ethnicity, sexuality, disability and social, political or financial status.

**Fig. 2.**
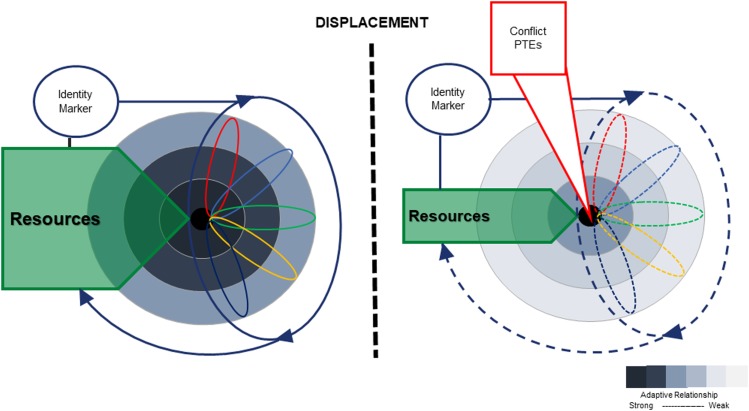
The impact of conflict and displacement on adaptive processes and resources. Note: The four environmental layers are depicted in concentric circles before and after displacement. The darkness of the shading indicates the strength of the adaptive relationship between the individual and their environment. Adaptive relationships are stronger for layers more proximal to the individual. Prior to displacement, individuals have developed strong adaptive links through the process of niche construction. Along with identity markers (such as gender, age, social status, class, ethnicity, disability and history of PTEs), this shapes their access to a pool of resources. Following displacement, the individual is literally removed from their environment, resulting in weakened adaptive relationships with the environmental layers. This disrupts the process of niche construction, such that previously adaptive behaviours may no longer succeed in securing access to resources. In addition, the pool of resources is diminished. Finally, the impact of conflict, human rights violations and PTEs also impact on adaptive systems at all layers.

Figure 2 depicts how adaptive processes are affected by displacement. In environment 1, adaptive relationships to the environmental layers are strong. However, following displacement, in a new transitional environment, these relationships are weakened and relationships to the new environment are yet to be strengthened. In addition, the available pool of resources is diminished. As a result, existing adaptive processes are forced to change, either because they are fractured by physical displacement, or because previously adaptive behaviours no longer function to promote access to resources. The impact of PTEs is noted, as this can further undermine adaptive capacities. Finally, the moderating impact of identity markers is noted, in that the impact of displacement on adaptive function and behaviour may differ depending on these markers.

The aim of this paper is to further develop the applicability of the ecological model we outline here to the perceived needs and wellbeing of Syrian refugees living in Jordan. We test this model against findings from a grounded theory analysis of perceptions of psychosocial concepts revealed across iterative interviews with key informants in psychosocial organisations supporting the Syrian refugee community in Jordan. In particular, we explore *karama* (dignity), a key concept relating calls for political freedom to personal and collective dignity in the Syrian uprising (Abbas, 2014) and the Arab Spring (West, 2011). We demonstrate how the broader range of factors included in an ecological perspective more closely match concepts which have arisen in the Syrian refugee community in response to the current crisis.

## Methods

Interviews were conducted in Amman, the capital city of Jordan, in three waves including December 2013–January 2014, August 2015–January 2016 and August–December 2016, as part of an iterative grounded theory project. The data presented here were part of a larger participatory action research project applying a grounded theory methodology (Strauss & Corbin, 1998; Charmaz, 2014) to generate theories regarding community explanatory models of distress; phenomenology of local concepts of mental health; barriers and facilitators supporting community uptake of psychosocial services; and discourse analysis of gendered constructions of identity in this population group. The theoretical model tested was developed through a review of the literature conducted concurrently with this project.

### Participants

Key informants within the Syrian refugee community in Jordan were purposively sampled over 3 years and were recruited through psychosocial organisations in Amman. Key informants are individuals who are knowledgeable about relevant attitudes and issues within a community (Thurman *et al*. 2007). Participants were 29 Syrians and Jordanians, working in psychosocial organisations supporting the Syrian refugee community, for at least 6 months, and were knowledgeable about local cultural values and norms. All but two of the Syrian participants were recently displaced. See Supplementary Table S1 for participant gender, nationality and profession. The study was approved by the Sydney University Human Research Ethics Committee (Institutional Review Board) (Project No. HREC 2013/803 and HREC 2015/148) and all participants provided informed consent.

### Interviews

The 27 interviews (two conducted in pairs) were structured according to an iterative approach, changing in response to the developing models and findings from previous interviews. See Supplementary Table S2 for example questions. In the first wave (December 2013–January 2014), eight initial interviews commenced with the semi-structured Community Readiness Model interview (Plested *et al*. 2009) which explored community attitudes to and readiness to address mental health consequences of displacement (Wells *et al*. 2018). This was followed by nine interviews (January 2014) exploring community explanatory models of distress. In the second wave of five interviews (August 2015–January 2016), an emergent model of how dignity is impacted by help seeking was presented to interviewees. The model was subsequently modified as participants explained how gender moderated constructions of dignity. In the third wave of four interviews (August–December 2016), the modified model was presented to interviewees until saturation was reached. Interviews were conducted by the first author, an Australian, female, doctoral student in clinical psychology.

### Data analysis

Grounded theory analysis (Strauss & Corbin, 1998; Charmaz, 2014) was undertaken by RW, DW and CL. Transcribed interviews were coded using *QSR nvivo* (version 11) qualitative analysis software (NVivo, 11, 2016). A subset of interviews was open coded by individual analysts until a coding system was agreed upon. Following cross-coding, axial coding was conducted, employing sensitising questions (Charmaz, 2014) to generate alternative explanations and hypotheses. Model development was focused on the identification of processes linking key concepts. Developing models were discussed with all the other authors during model development. An iterative process was employed to review data, using constant comparison to compare the model to raw data at each stage of interviewing and model development. Member checking was conducted by asking subsequent interviewees for feedback on the emergent model which was then modified and presented to more interviewees.

## Results

We now present exemplars of the themes emerging from the analysis. Two key Arabic language terms emerged. We explore the social processes associated with these phenomena, including gender, identity, interpersonal relationships, safety, justice and their relationships to resources.

### Karama (dignity, 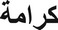)

*Karama* (dignity) was described as central to Syrian life, connected to identity and justice, and as a foundation for social exchange. Without basic rights and resources, one's access to *karama* may be seriously undermined.

‘*Karama means, I'm a human.*’ Syrian male volunteer

‘*Because here, for the people, the most important thing is karama. If I lose my karama, I lose myself.*’ Female protection worker

Karama was described as integral to identity, in relation to self, family, social standing and culture.

‘*The Syrian women here in Jordan, they have their identity, the Syrian identity, “I will just teach my children to present the Syrian way for everyone.” This is a part of their karama.*’ Female psychologist

Three key qualities and behaviours were associated with how *karama* was described.

1. Patience and strength were described as virtues and as ways of coping.

‘*It*'*s like being patient. Be patient because you will have a reward from Allah. Or being strong. Be very strong to face these hard things that happened.*’ Male psychologist

‘*And if someone survived torture, they wouldn*'*t take him to the psychologist, they would say “You're a man, be tough. If you take this, you will go to heaven.”*’ Male volunteer

However, social norms regarding patience were described as different for women.

‘*[women need] Resilience, definitely. Patience also, but in a different way than men. I think that*'*s because she would be sacrificing a lot, from what I have seen.*’ Female psychologist

1. Self-reliance was described as a necessary component of *karama.*

‘*A man would find it so hard to say I need help, I need money. It*'*s difficult for men to say something like that.*’ Female psychologist

However, this may not apply in the same way to women.

‘*[for women] Well, maybe not self-reliance, because she relies on her husband a lot.*’ Female psychologist

1. Refusing things was described as a way to demonstrate self-reliance through:
  - Refusing help

‘*Many of them…refused to go to any organisation for help and assistance. And even when we used to go to them and tell them if you want anything we will provide it to you, they wouldn't say anything*.’ Male volunteer

1. Refusing to participate in activities which contravene one's values.

‘*They understand karama in that they had many offers in Syria to have a good life, to avoid all this trauma, just by doing something against their rules. But they didn*'*t. So*… *where ever you are, you have your karama.*’ Female psychologist

Refusal was also affected by gender as participants explained that open refusal and resistance is often a path that is not open to women because of the threat of violence from men.

‘*A women, head on, cannot resist. It is a disaster*… *because that pathway contains lots of danger or potential catastrophes. If every woman tries to reject or resist, each woman would need at least one policeman to walk with to protect her.*’ Female protection worker

In addition to these behavioural components, *karama* was described as a socially determined phenomenon, defined in interpersonal context and in relation to providing for one's family.

‘*It gets you more recognition, it gets you more respect from others, I think the way people look at you…* *No one can live alone, because we are social beings.*’ Female psychologist

‘*The feeling of taking care of my family and I can*'*t offer them any money or food, or I can*'*t give them the basic things that they need, this can decrease karama.*’ Male physiotherapist

### Sudme (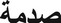)

The word *sudme* (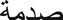) was commonly used in reference to the emotional impact of the crisis. *Sudme* may often be translated into English as trauma, although in lay usage it is not linked to psychological pathology. Rather, it is a normal reaction to an extreme situation.

‘*It has a spoken meaning which is different than for psychologists*…*When they say sudme, it means I'm shocked, reaching to the definition of what trauma is…* *I went through something very hard to go through and right now I can*'*t handle everything in my life. That*'*s how they explain it.*’ Female psychologist

*Sudme* is much broader than the English word trauma. It refers to PTEs and the whole refugee experience, including the accumulation of day-to-day stressors which were often described as pressure.

‘*It*'*s not the shock anymore, it*'*s like what is the result of all of that. Like feeling less than other people, feeling less dignity and they have a saying in the Syrian language…* *It*'*s like, when you leave your area, you will be of less dignity*.’ Female psychologist

‘*Adughat [pressure]. A lot of Syrians they are aware that most of their uncomfortable feelings, or negative feelings, are not related to trauma itself, but to the pressure of the stress they have here in the new community, the lack of services, lack of opportunities, lack of social support. All that make them more stressed. It*'*s a very common [term]*… *they refer to the current situation, not only for the traumatic event itself.*’ Female psychologist

Responses to *sudme* may include intense distress, hopelessness, somatic difficulties and a sense of overwhelming pressure. The emotional impact of PTEs and the accumulation of everyday stressors cannot be disentangled from one another.

‘*First, they speak about what they are suffering: difficulty in sleeping, difficulty in coming out of the house and facing people, and for the man, difficulty in how they will feed his family. And when he thinks about this, he feels like his head is going to explode from the all that he has.*’ Male religious scholar

*Sudme* may undermine the social components of *karama* when *sudme* leads to shame.

‘*It*'*s not acceptable what I mention*…*about the traumatic event. Do you know why? Because many times it*'*s related to humiliation, it*'*s related to shame, it*'*s related to breaking their karama*… *They can*'*t answer this question, why this happened to them?*’ Male psychologist

*Sudme* was described as undermining *karama* and impacting on interpersonal and familial relationships.

‘*[many men say] I don*'*t have any dignity. I'm here and I don*'*t have anything*… *the NGOs are controlling my life, not me*…*All of that helps them to feel less karama.*’ Female psychologist

‘*To them it*'*s only weakness and they can*'*t accept being weak* …*“If I had money, I would be able to sleep at night, I wouldn*'*t beat my sons.”*’ Female psychologist

### Impact of gender

A key issue which emerged, was how both *karama* and the impact of *sudme* were affected by gender.

Participants described how women often do not have direct access to *karama*.

‘*Well it*'*s defined because usually in these cultures women are followers, she gets her karama, she takes it from her man.*’ Female psychologist

This was discussed in relation to women being in a position of dependence.

‘*For the abused girl or wife it*'*s related to karama*… *a lot of them, honestly, they prefer to keep silent*…*Because of fearing from the culture issues*… *because they are already dependent to the husband.*’ Female psychologist

As a result, women are required to be flexible to the needs of the people they depend on.

‘*Before the crisis*… *men were presenting their rules, their orders “Don*'*t understand it. Be flexible for it. Just do it.”*… *“Be dependent.”*’ Female psychologist

However, displacement has led to an interchange of roles because it is acceptable for women to seek support, but not men.

‘*The woman is the one who gets all the needs of the family satisfied, but not in the way she used to before, more independently*… *She*'*s starting to not exactly replace, but she*'*s taking some of the roles that the father used to have back in Syria*… *perhaps right now she is the one who takes the money, so she has the money in her hand.*’ Female psychologist

### Grounded theory model

**Fig. 3.**
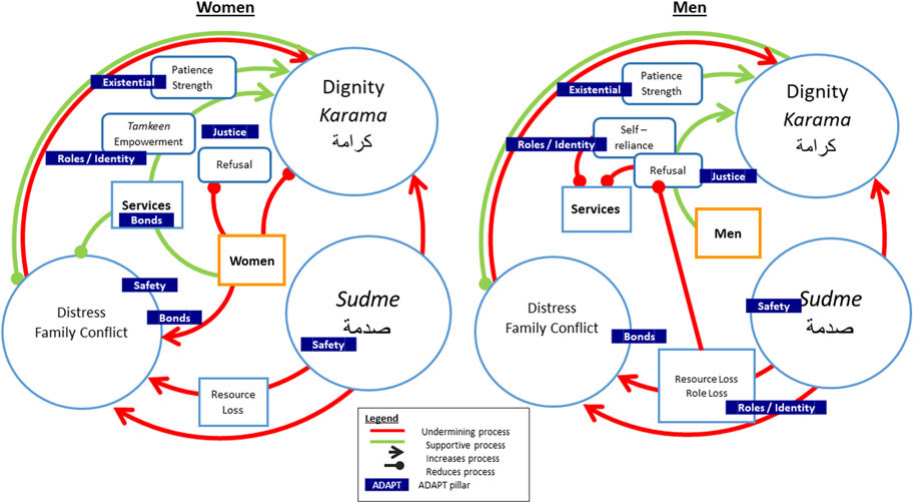
Niche construction: processes that undermine (sudme) or promote (karama) wellbeing are moderated by gender.

Figure 3 displays a model emerging from the above findings. The model depicts how *karama* and *sudme* interact with one another to impact adaptation, represented by processes that contribute to or alleviate distress and family conflict (here after distress). There are separate models for men and women, representing the moderating impact of gender.

For men, *karama* is increased by acts of refusal and self-reliance and the qualities of patience and strength. *Karama* is undermined directly by *sudme* (e.g. by PTEs, such as torture). *Sudme* leads directly to distress but is also amplified by resource and role loss. In addition, refusal and self-reliance mean that men are less likely to access support services, blocking an avenue to alleviation of distress. Finally, distress undermines *karama*. Conversely, *karama* reduces distress.

For women, *karama* is also undermined directly by sudme, leading to distress, amplified by resource loss. Patience and strength may increase *karama*, but there is not a direct pathway to *karama* for women through refusal and self-reliance, which are often not an option. Instead, women have greater access to services which may both directly reduce distress and indirectly increase *karama* by providing opportunities for empowerment and increasing resources for patience and strength.

### Comparing the grounded theory model and the ecological model

**Fig. 4.**
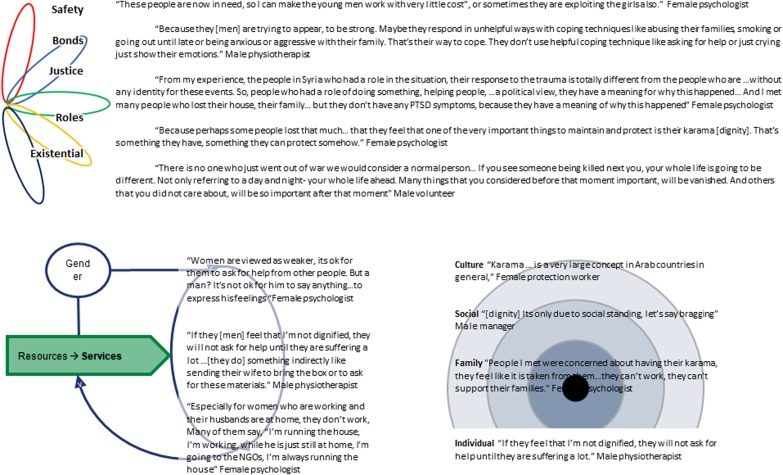
Components of the ecological model compatible with the grounded theory model.

We will now consider the components of the ecological model and how they relate to the grounded theory model. See Fig. 4 for quotes relating to the ecological model.

#### ADAPT pillars

Figure 3 highlights areas in the grounded theory model which are related to the ADAPT pillars (Silove, 2013). For example, refusal may be connected to justice when it involves maintaining *karama* by rejecting things which are not consistent with personal values. Ideals of patience and strength may be connected to existential meaning through religion. Self-reliance, role loss and *karama* are all connected to roles and identity in that they provide ways for people to enact identity. *Sudme* is a core concept which is clearly connected to the safety system through its relationship with trauma. Finally, distress and family conflict have direct bearing on interpersonal bonds, while *karama* is partly defined by social affiliations. Of note, this model also highlights the interconnected nature of the ADAPT pillars in adaptive functioning.

#### Ecological layers

*Karama* can be conceptualised at the individual level (promoting personal wellbeing); is often interpersonally determined by how one cares for one's family; can operate as a form of social capital, demonstrating a person's social standing, for example, through enactments of refusal; and is often discussed as a phenomenon specific to Syrian culture. Consideration of all these levels can help to uncover how the concept may operate to determine access to resources. For example, cultural norms for men may lead to refusal, supporting personal *karama*, but reducing resources for the family, leading to increased distress and family conflict.

Similarly, *sudme* can be thought of as a personal reaction to trauma; a phenomenon which impacts on family functioning; a collective social crisis impacting access to rights and resources; and a culture bound idiom of distress.

#### Niche construction

We now consider how a change in environment impacts on the adaptive functions of behaviours and consider how gender moderates the process of niche construction, where individuals respond to environmental demands and thereby change their environment.

#### In Syria

For men living in Syria before the crisis, access to resources enabled the performance of male gender norms of patience, self-reliance and resistance. These were adaptive in promoting *karama* for the self and family, increasing access to further resources. For women living in Syria before the crisis, access to resources was often restricted or filtered through men. As women were perceived as dependent, less self-reliance was expected of them than men. When women do not have direct access to safety and resources, resistance may be counter-productive and put them in danger of abuse. Thus, behavioural flexibility tended to be more adaptive than resistance, as a diverse repertoire of behaviours and coping skills would be needed to access resources (i.e. a broader niche).

#### In displacement

In displacement, for all genders, family access to resources through employment or social connections is diminished. Distress may overwhelm personal coping resources making patience and strength increasingly difficult.

For men, resource loss over time undermines the previously adaptive function of *karama* promoting behaviours because resource loss makes self-reliance increasingly difficult and resistance may undermine access to the very resources which could promote adaptation. This loss is amplified by distress, which undermines personal coping resources needed for resilience.

For women, social acceptability of seeking services to address basic needs gives women direct access to resources such as financial, social, psychological, educational and capacity-building. Access to these resources also reduces distress, promoting future resilience.

Female social norms, which include a broader niche or need for greater flexibility in adaptive functions, enable women to take advantage of resources available in the displacement environment, increasing their adaptive capacities. This, in turn, impacts the social system by disrupting gender roles. In contrast, male norms of self-reliance and resistance prevent them from taking advantage of these resources in the same way. As a result, participants described how many women were taking advantage of services which would have been stigmatised in the past, such as mental health services. In contrast, many men were struggling with loss of role, while norms of resistance mean that men must pay a price (their *karama*) for asking for help.

## Discussion

The ecological model presented here resonates with concepts of wellbeing described by Syrian refugees in Jordan, both in the current analysis and a previous systematic review of psychosocial needs assessments (Wells *et al*. 2016*a*). Although previous theoretical models have outlined ecological approaches to refugee psychosocial health (Ryan *et al*. 2008; Silove, 2013; Drozdek, 2015; Miller & Rasmussen, 2017), this paper applies these concepts to an in-depth grounded-theory analysis of the ongoing Syrian crisis, and explicitly examines the transactional nature of issues raised by members of the Syrian refugee community across three waves of interviewing over the periods 2013–2016. The Syrian community concepts of *karama* and *sudme* demonstrate how the adaptive function of values and behaviour can change substantially with a major change in the environment. The concept of niche construction provides an important framework for understanding how some members of a community will be better placed to respond to and take advantage of the changed environmental circumstances to support their own adaptive function while contributing to changes in their social world. The findings confirm the congruence of the ADAPT concepts with the narrative of key informants working with Jordan's Syrian refugee community.

An ecological approach goes beyond a cultural formulation which adapts imported therapeutic models to local explanatory models (Kirmayer, 2006). Rather, an ecological model calls for a formulation which considers the interplay of social and cultural factors. By identifying how agency is represented in the concept of *karama*, we demonstrate how changes in environment can open up spaces for shifts in agency and power at both individual and collective levels in a way that has empowered some women in the displacement environment (Smith, 2008). Identifying patterns of adaptive behaviour taken up by women does not negate the impact of gendered power relations and violence on women's wellbeing. Worldwide, prevalence rates of anxiety and depression are higher among women, while substance use is higher among men (Steel *et al*. 2014). Connell's (2005) concept of hegemonic masculinity offers a useful frame to consider *karama*. The concept of *karama*, as described in this study, offers a masculinised ideal that is challenging to embody, especially as displacement has undermined access to resources. The distress caused by failure to embody this ideal, along with attitudes that limit men's avenues for acknowledging this distress, may lead to problems for all involved. This is consistent with our previous findings in the Syrian refugee community in Jordan where women identified increases in family violence with men's loss of role (Wells *et al*. 2016*a*).

## Applying this ecological model in clinical and research practice

An ecological analysis can guide community-level preventative interventions which promote adaptation. For example, research in another post-conflict setting has demonstrated how intimate partner violence leads to distress and explosive anger attacks among women, leading to harsh parenting practices (Rees *et al*. 2015). Our model would predict that providing new role opportunities to men in a way which supports *karama* would improve personal wellbeing, as well as reducing conflict and violence among all family members. Given that reporting of and help seeking for mental health difficulties may be stigmatised in the Syrian community (Maziak *et al*. 2002; Gearing *et al*. 2013), especially among men (Wells *et al*. 2016*b*), outcomes could be measured by developing a locally validated measure of role adjustment and its relation to distress and family functioning. An intervention programme would likely be most effectively delivered at the community level.

International consensus calls for a greater emphasis on community-level interventions (IASC, 2007), yet methodologically sound evidence regarding their effectiveness is lacking (Nickerson *et al*. 2011). Current advancements in methodology, including latent variable modelling (Gilmore *et al*. 2016), stepped wedge design (Brown & Lilford, 2006), network analysis (Jayawickreme *et al*. 2017) and rigorous qualitative analysis (Pereira *et al*. 2007) could lead to considerable progress. We suggest that the model presented here provides an example of an approach that can develop operational and testable hypotheses regarding the role that social, cultural and interpersonal factors play in individual wellbeing among refugees. It provides a method to widen the scope of an individual clinical psychology formulation to explore possible causal and maintaining factors in a broader ecological system.

Threat remains current for many refugee communities who have not been resettled to situations of safety. In such a situation, fear-related responses, such as hyperarousal or cognitive bias towards threat-related stimuli, may not be symptoms of pathology, rather they are possibly adaptive responses aimed at preventing future danger (Silove, 1998). Theoretical approaches which recognise that threat of human rights violations remains constant, such as continuous traumatic stress (Straker, 2013), may offer more clinical utility and could direct resources to modifiable ongoing risk factors (Steel *et al*. 2006; Miller & Rasmussen, 2014) or target cognitive mechanisms which help people cope with these realities, for example, through enhancing self-efficacy (Brown *et al*. 2016). The PM + programme (Rahman *et al*. 2016), which focuses on problem-solving skills to address practical psychosocial issues, may offer a useful framework for reducing distress. One of us (OSY) is currently working to train community psychosocial workers in Northern Syria to employ an ecological focus.

## Limitations

This research may have benefitted from an intersectional lens from the outset (Crenshaw, 1991). Such an approach recognises that the impact of identity markers such as gender, class, sexuality or ethnicity may interact and intersect; thus, attending only to one (e.g. gender) may ignore the impact of others (e.g. ethnicity). The focus on gender which emerged during the interviews reflects the issues as raised by community members themselves, which included a binary definition of gender. Such a definition may not represent the experience of all. The issues raised regarding gender-based violence would have benefitted from a more in-depth, critical approach. We would like to caution against an interpretation of these findings that situates violence against women within an orientalist discourse (Said, 1978) that draws attention to the problem of sexism in Arabic-speaking cultures, while ignoring that violence against women exists in all cultures (Abu-Lughod, 2013). We acknowledge the fluid nature of culture (Kirmayer, 2006) within historical context and that individual negotiations with *karama* will differ across individuals and settings. We also note that the narratives of change discussed here may have been influenced by the social identity of interviewees, who were psychosocial activists invested in a system of humanitarian action and exposed to care-seeking individuals. As well-educated individuals, their comments may represent the views of a particular class of Syrian society and cannot be generalised to the community as a whole [discussed in greater detail in Wells *et al*. (2016*b*)]. We chose to interview individuals with some professional training as their familiarity with psychosocial issues would provide them with skills for coping with distress and stigma associated with the interview topics. The interviewer was a white, female psychologist from Australia. Her identity may have been a barrier to some participants who may have found it difficult to trust that she would understand and identify with their lived experience and culture or felt that they needed to present a specific image to outsiders. However, this may also have been a strength, as her lack of familiarity with the culture meant that participants needed to explicitly describe concepts, rather than rely on shared understanding.

## Conclusions

We hope this model can contribute to the formulation of interventions and research questions which help to address the broad range of ecological factors raised by community members in this study. Syria has a high rate of tertiary education (including for women) and many skilled professionals. Research can examine whether their skills are being put to use in the new environment or whether their political status precludes this, as well as what support community leaders, teachers, journalists, religious leaders and engineers need to build capacity in their communities? When assessing the outcomes of programmes, we can ask whether niche construction is achieving its end by examining whether programmes have increased access to further resources including material outcomes (such as increased access to employment) or psychosocial outcomes (such as increased social capital). We can use this approach to ensure that programmes consider all the nested layers of the environment. We hope that such an approach will support the adaptive capacities of communities to come together to rebuild and recover.

## Supplementary material

For supplementary material accompanying this paper visit http://dx.doi.org/10.1017/gmh.2018.30. click here to view supplementary material
