# Supplementary material for: An ecological model of adaptation to displacement: individual, cultural and community factors affecting psychosocial adjustment among Syrian refugees in Jordan
Source: Glob Ment Health (Camb). 2018 Dec 20;5:e42. doi: 10.1017/gmh.2018.30 (PMC6315281; doi:10.1017/gmh.2018.30)
Supplement: Supplementary file 1 [file S2054425118000304sup001.docx]

|  | ID | Gender | Nationality | Profession |
| --- | --- | --- | --- | --- |
| 2013- | 1 | M | Syrian | Relief Worker (volunteer) |
| 2014 | 2 | M | Syrian | Psychologist |
|  | 3 | F | Syrian | Psychologist |
|  | 4 | F | Syrian | Manager psychosocial organisation |
|  | 5 | F | Jordanian | Psychologist |
|  | 6 | M | Syrian | Psychologist |
|  | 7 | M | Jordanian | Psychologist |
|  | 8 | F | Syrian | Teacher |
|  | 9 | M | Syrian | Relief Worker (volunteer) |
|  | 10 | M | Syrian | Psychologist |
|  | 11 | F | Syrian | Psychologist |
|  | 12 | M | Syrian | Religious activist |
|  | 13 | M | Jordanian | Psychologist / religious scholar |
|  | 14 | M | Syrian | Psychologist |
|  | 15 | M | Syrian | Activist (psychosocial volunteer) |
|  | 16 | M | Syrian | Manager psychosocial organisation |
|  | 17 | M | Syrian | Relief Worker (volunteer) |
|  | 18 | F | Syrian | Activist (psychosocial volunteer) |
|  | 19 | F | Jordanian | Psychologist |
|  | 20 | M | Syrian | Medical Doctor |
|  | 21 | M | Syrian | Psychologist |
|  | 22 | M | Syrian | Psychologist |
| 2015 | 23 | F | Jordanian | Psychologist |
|  | 24 | F | Jordanian | Protection |
|  | 25 | F | Jordanian | Protection |
| 2016 | 26 | F | Syrian | Psychologist |
|  | 27 | F | Jordanian | Psychologist |
|  | 28 | M | Jordanian | Physiotherapist |
|  | 29 | F | Jordanian | Psychologist |

**Supplementary Table 1. Key informants’ gender, nationality and profession**

**Supplementary Table 2. Example interview questions**

| **Interview wave** | **Example questions** |
| --- | --- |
| **2013-2014** | To what extent are mental health concerns viewed as problem within the Syrian refugee community?  How are mental health concerns perceived within the community?  To whom would an individual affected by mental health problems turn to first for help in the Syrian refugee community? Why?  What are the primary barriers to accessing services in the Syrian refugee community? What do Syrian Refugees think causes Mental Health concerns?  How would a Syrian refugee describe to a friend or family member how a mental health concern developed?  What are some of the coping strategies Syrian refugee families use to manage Mental Health concerns? How does being a refugee impact on care seeking?  How would having a mental health problem impact upon someone’s role in the family? |
| **2015** | Presentation of initial model depicting how gender moderates impact on care seeking was presented Descriptions of *karama* and *sudme* were presented  Participants indicated that description of *karama* described concepts generally related to men and not women. Follow-up questions probed the nature of *karama* for women. |
| **2016** | Presentation of updated model including impact of gender on adaptive processes in displacement  How is *karama* understood differently by men and women?  What attributes or behaviours would be associated with *karama* for women?  How are these attributes or behaviours related to access to resources for women and men? |
